# REGULATION OF NEURONAL MITOCHONDRIAL BIOENERGETICS BY LIPID METABOLITES UPREGULATED IN DEMENTIA

**DOI:** 10.1093/geroni/igac059.1374

**Published:** 2022-12-20

**Authors:** Stephanie Heimler, K Allison Amick, Jaclyn Bergstrom, Mohit Jain, Anthony J Molina

**Affiliations:** UC San Diego School of Medicine, San Diego, California, United States; Wake Forest School of Medicine, Winston Salem, North Carolina, United States; UC San Diego School of Medicine, San Diego, California, United States; UC San Diego School of Medicine, San Diego, California, United States; UC San Diego School of Medicine, San Diego, California, United States

## Abstract

Mitochondrial dysfunction occurs early in Alzheimer’s disease (AD) progression and is evident in the Central Nervous System (CNS) and peripheral circulating cells. While there is evidence indicating that bioenergetic decline can drive the early pathogenesis of AD, little is known about the extracellular factors that alter neuronal bioenergetic capacity. We hypothesized that circulating metabolites contribute to neuronal bioenergetic decline associated with AD. Using a cohort comprised of participants with normal cognition, mild cognitive impairment, and dementia, we show that human serum harbors circulating, non-cellular factors capable of mediating neuronal bioenergetic differences according to the cognitive status of the serum donor. We developed a novel screening-based approach to identify candidate “mito-active” lipid metabolites in human serum that could mediate differences in bioenergetic capacity. Among these, Nervonic Acid and 15-epi-PGA1 were predicted to be mitochondrial inhibitors upregulated in participants with dementia. Neurons exposed to physiologically-relevant ranges of these molecules in-vitro exhibited a dose-dependent reduction in maximal mitochondrial respiration. We found that 500ug/mL of Nervonic Acid and 9ug/mL of 15-epi-PGA1 reduced maximal mitochondrial respiration by 62.3% and 63.3%, respectively, thereby validating our screening and prediction approach. Future experiments may be directed towards investigating if and how these and other mito-active metabolites of interest cross the blood-brain barrier to meaningfully affect AD. Furthermore, identified mito-active molecules can be investigated in other clinical cohorts to examine their role/s in multiple age-related or neurological conditions. This work expands our mechanistic understanding of how extrinsic factors associated with age and cognitive status contribute to neuronal bioenergetic decline.

